# 1-(4-Chloro­phen­yl)-3-(5-methyl-2-fur­yl)prop-2-en-1-one

**DOI:** 10.1107/S1600536809044456

**Published:** 2009-10-31

**Authors:** Huan-Mei Guo

**Affiliations:** a Microscale Science Institute, Weifang University, Weifang 261061, People’s Republic of China

## Abstract

The title compound, C_14_H_11_ClO_2_, was prepared from 4-chloro­hypnone and 5-methyl­furfural by an aldol condensation reaction. The dihedral angle formed between the two benzene rings is 7.71 (2)°. The crystal structure is stabilized by C—H⋯O inter­actions.

## Related literature

For the biological activity of chalcones, see: Anto *et al.* (1994 ▶); Dimmock *et al.* (1998 ▶); Hsieh *et al.* (1998 ▶); De Vincenzo *et al*. (2000 ▶). For a related structure, see: Guo *et al.* (2008 ▶).
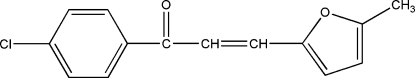


## Experimental

### 

#### Crystal data

C_14_H_11_ClO_2_
*M* *_r_* = 246.68
Monoclinic, 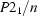

*a* = 8.350 (3) Å
*b* = 15.732 (5) Å
*c* = 9.660 (3) Å
β = 106.882 (5)°
*V* = 1214.4 (6) Å^3^
*Z* = 4
Mo *K*α radiation
μ = 0.30 mm^−1^
*T* = 273 K
0.50 × 0.30 × 0.25 mm

#### Data collection

Bruker SMART CCD area-detector diffractometer
Absorption correction: none
7894 measured reflections
2988 independent reflections
2055 reflections with *I* > 2σ(*I*)
*R*_int_ = 0.018

#### Refinement

*R*[*F* ^2^ > 2σ(*F* ^2^)] = 0.054
*wR*(*F* ^2^) = 0.180
*S* = 1.07
2988 reflections
154 parameters
H-atom parameters constrained
Δρ_max_ = 0.32 e Å^−3^
Δρ_min_ = −0.36 e Å^−3^

### 

Data collection: *SMART* (Bruker, 1997 ▶); cell refinement: *SAINT* (Bruker, 1997 ▶); data reduction: *SAINT*; program(s) used to solve structure: *SHELXS97* (Sheldrick, 2008 ▶); program(s) used to refine structure: *SHELXL97* (Sheldrick, 2008 ▶); molecular graphics: *SHELXTL* (Sheldrick, 2008 ▶); software used to prepare material for publication: *SHELXTL*.

## Supplementary Material

Crystal structure: contains datablocks global, I. DOI: 10.1107/S1600536809044456/tk2555sup1.cif

Structure factors: contains datablocks I. DOI: 10.1107/S1600536809044456/tk2555Isup2.hkl

Additional supplementary materials:  crystallographic information; 3D view; checkCIF report

## Figures and Tables

**Table 1:** Hydrogen-bond geometry (Å, °)

*D*—H⋯*A*	*D*—H	H⋯*A*	*D*⋯*A*	*D*—H⋯*A*
C9—H9*A*⋯O1^i^	0.93	2.53	3.380 (4)	153
C14—H14*A*⋯O1	0.93	2.47	2.795 (2)	100

Symmetry code: (i) 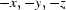.

**Fig. 1.**
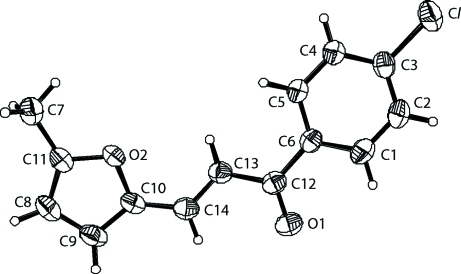
The molecular structure of (I) with the atom-labeling scheme. Displacement ellipsoids are drawn at the 30% probability level.

## supplementary crystallographic information

### Comment

Among flavonoids, chalcones have been identified as interesting compounds with multiple biological actions, including anti-inflammatory (Hsieh *et al.*, 1998) and anti-oxidant (Anto *et al.*, 1994) activities. Of particular interest is the effectiveness of chalcones against cancer (De Vincenzo *et al.*, 2000; Dimmock *et al.*, 1998). As part of our search for new biologically active compounds, we synthesized the title chalcone, (I), and report its crystal structure herein.

The molecular structure of (I), Fig. 1, comprises a furan ring and a chlorophenyl group. These groups are not co-planar as seen in the value of the dihedral angle between them of 7.71 (2)°. Bond distances and angles conform to literature precedents (Guo, *et al.*, 2008). There are intre- and inter-molecular C—H···O interactions that stabilize the molecular and crystal structures, respectively (Table 1). The intermolecular contacts lead to the formation of centrosymmetric dimers.

### Experimental

Compound (I) was prepared in 80% yield by stirring an ethanol (30 ml) mixture comprising 4-chlorohypnone (0.02 mol), 5-methylfurfural (0.02 mol) and 10% NaOH  (10 ml) for 3 h. Single crystals were obtailed by recrystallization of (I) from ethyl acetate at room temperature.

### Refinement

H atoms were positioned geometrically and allowed to ride on their parent atoms, with C—H distances of 0.93–0.96 Å, and with *U*_iso_(H) = 1.2–1.5*U*_eq_(C).

### Crystal data

**Table:**

C_14_H_11_ClO_2_	*F*(000) = 512
*M**_r_* = 246.68	*D*_x_ = 1.349 Mg m^−^^3^
Monoclinic, *P*2_1_/*n*	Mo *K*α radiation, λ = 0.71073 Å
Hall symbol: -P 2yn	Cell parameters from 2055 reflections
*a* = 8.350 (3) Å	θ = 2.6–28.4°
*b* = 15.732 (5) Å	µ = 0.30 mm^−^^1^
*c* = 9.660 (3) Å	*T* = 273 K
β = 106.882 (5)°	Block, yellow
*V* = 1214.4 (6) Å^3^	0.50 × 0.30 × 0.25 mm
*Z* = 4	

### Data collection

**Table:**

Bruker SMART CCD area-detector diffractometer	2055 reflections with *I* > 2σ(*I*)
Radiation source: fine-focus sealed tube	*R*_int_ = 0.018
graphite	θ_max_ = 28.4°, θ_min_ = 2.6°
φ and ω scans	*h* = −10→11
7894 measured reflections	*k* = −20→21
2988 independent reflections	*l* = −11→12

### Refinement

**Table:**

Refinement on *F*^2^	Primary atom site location: structure-invariant direct methods
Least-squares matrix: full	Secondary atom site location: difference Fourier map
*R*[*F*^2^ > 2σ(*F*^2^)] = 0.054	Hydrogen site location: inferred from neighbouring sites
*wR*(*F*^2^) = 0.180	H-atom parameters constrained
*S* = 1.07	*w* = 1/[σ^2^(*F*_o_^2^) + (0.0853*P*)^2^ + 0.305*P*] where *P* = (*F*_o_^2^ + 2*F*_c_^2^)/3
2988 reflections	(Δ/σ)_max_ < 0.001
154 parameters	Δρ_max_ = 0.32 e Å^−^^3^
0 restraints	Δρ_min_ = −0.36 e Å^−^^3^

### Special details

**Table:**

Geometry. All e.s.d.'s (except the e.s.d. in the dihedral angle between two l.s. planes) are estimated using the full covariance matrix. The cell e.s.d.'s are taken into account individually in the estimation of e.s.d.'s in distances, angles and torsion angles; correlations between e.s.d.'s in cell parameters are only used when they are defined by crystal symmetry. An approximate (isotropic) treatment of cell e.s.d.'s is used for estimating e.s.d.'s involving l.s. planes.
Refinement. Refinement of *F*^2^ against ALL reflections. The weighted *R*-factor *wR* and goodness of fit *S* are based on *F*^2^, conventional *R*-factors *R* are based on *F*, with *F* set to zero for negative *F*^2^. The threshold expression of *F*^2^ > σ(*F*^2^) is used only for calculating *R*-factors(gt) *etc*. and is not relevant to the choice of reflections for refinement. *R*-factors based on *F*^2^ are statistically about twice as large as those based on *F*, and *R*- factors based on ALL data will be even larger.

### Fractional atomic coordinates and isotropic or equivalent isotropic displacement parameters (Å^2^)

**Table:**

	*x*	*y*	*z*	*U*_iso_*/*U*_eq_	
Cl	0.67915 (10)	0.11664 (5)	0.96441 (7)	0.0964 (3)	
O2	0.29242 (17)	0.21735 (9)	0.00449 (14)	0.0596 (4)	
C14	0.1656 (3)	0.10736 (14)	0.1064 (3)	0.0658 (5)	
H14A	0.0836	0.0655	0.0908	0.079*	
C13	0.2634 (3)	0.11657 (13)	0.2411 (2)	0.0626 (5)	
H13A	0.3490	0.1566	0.2598	0.075*	
C10	0.1736 (3)	0.15490 (15)	−0.0158 (2)	0.0629 (5)	
C12	0.2409 (3)	0.06591 (13)	0.3607 (3)	0.0650 (5)	
C5	0.4834 (3)	0.13765 (16)	0.5410 (2)	0.0710 (6)	
H5A	0.5052	0.1688	0.4666	0.085*	
C6	0.3508 (3)	0.08210 (12)	0.5095 (2)	0.0587 (5)	
C4	0.5843 (3)	0.14810 (17)	0.6800 (3)	0.0774 (7)	
H4A	0.6746	0.1854	0.6994	0.093*	
O1	0.1329 (2)	0.01152 (11)	0.3404 (2)	0.0909 (6)	
C3	0.5513 (3)	0.10340 (14)	0.7892 (2)	0.0670 (6)	
C11	0.2738 (3)	0.25576 (15)	−0.1251 (2)	0.0653 (6)	
C2	0.4203 (4)	0.04941 (17)	0.7630 (3)	0.0860 (8)	
H2A	0.3984	0.0195	0.8386	0.103*	
C9	0.0819 (3)	0.15581 (19)	−0.1574 (3)	0.0800 (7)	
H9A	−0.0076	0.1203	−0.2009	0.096*	
C8	0.1463 (3)	0.21949 (18)	−0.2252 (3)	0.0783 (7)	
H8A	0.1079	0.2343	−0.3224	0.094*	
C1	0.3202 (4)	0.03911 (16)	0.6244 (3)	0.0844 (8)	
H1A	0.2293	0.0023	0.6067	0.101*	
C7	0.3947 (3)	0.32399 (17)	−0.1277 (3)	0.0853 (7)	
H7A	0.4700	0.3315	−0.0324	0.128*	
H7B	0.3355	0.3761	−0.1594	0.128*	
H7C	0.4572	0.3086	−0.1932	0.128*	

### Atomic displacement parameters (Å^2^)

**Table:**

	*U*^11^	*U*^22^	*U*^33^	*U*^12^	*U*^13^	*U*^23^
Cl	0.1060 (6)	0.1184 (6)	0.0598 (4)	0.0105 (4)	0.0160 (4)	0.0124 (3)
O2	0.0562 (8)	0.0653 (8)	0.0492 (7)	0.0017 (6)	0.0026 (6)	−0.0025 (6)
C14	0.0611 (12)	0.0613 (12)	0.0731 (14)	−0.0025 (10)	0.0163 (10)	−0.0093 (10)
C13	0.0600 (12)	0.0571 (11)	0.0675 (13)	−0.0025 (9)	0.0137 (10)	−0.0028 (9)
C10	0.0532 (11)	0.0707 (13)	0.0586 (12)	0.0006 (9)	0.0068 (9)	−0.0124 (9)
C12	0.0682 (13)	0.0501 (11)	0.0779 (14)	−0.0017 (10)	0.0231 (11)	−0.0008 (10)
C5	0.0748 (14)	0.0776 (14)	0.0610 (12)	−0.0125 (12)	0.0201 (11)	0.0129 (11)
C6	0.0644 (12)	0.0462 (10)	0.0682 (12)	0.0024 (9)	0.0232 (10)	0.0047 (9)
C4	0.0774 (15)	0.0846 (16)	0.0666 (14)	−0.0147 (13)	0.0151 (11)	0.0133 (12)
O1	0.0971 (13)	0.0774 (11)	0.0948 (13)	−0.0329 (10)	0.0224 (10)	−0.0050 (9)
C3	0.0738 (14)	0.0675 (13)	0.0610 (12)	0.0142 (11)	0.0213 (10)	0.0084 (10)
C11	0.0633 (12)	0.0738 (13)	0.0535 (11)	0.0127 (10)	0.0088 (9)	−0.0016 (9)
C2	0.104 (2)	0.0835 (17)	0.0741 (16)	−0.0067 (15)	0.0314 (15)	0.0243 (13)
C9	0.0655 (13)	0.1002 (18)	0.0643 (13)	−0.0104 (13)	0.0028 (11)	−0.0208 (13)
C8	0.0738 (14)	0.1012 (18)	0.0504 (11)	0.0100 (13)	0.0030 (10)	−0.0061 (11)
C1	0.0927 (17)	0.0735 (15)	0.0897 (19)	−0.0194 (13)	0.0306 (15)	0.0162 (13)
C7	0.0932 (18)	0.0817 (16)	0.0752 (15)	0.0017 (14)	0.0154 (14)	0.0149 (13)

### Geometric parameters (Å, °)

**Table:**

Cl—C3	1.731 (2)	C4—C3	1.361 (3)
O2—C11	1.358 (3)	C4—H4A	0.9300
O2—C10	1.370 (3)	C3—C2	1.349 (4)
C14—C13	1.328 (3)	C11—C8	1.340 (3)
C14—C10	1.415 (3)	C11—C7	1.479 (4)
C14—H14A	0.9300	C2—C1	1.367 (4)
C13—C12	1.460 (3)	C2—H2A	0.9300
C13—H13A	0.9300	C9—C8	1.388 (4)
C10—C9	1.360 (3)	C9—H9A	0.9300
C12—O1	1.217 (3)	C8—H8A	0.9300
C12—C6	1.485 (3)	C1—H1A	0.9300
C5—C4	1.372 (3)	C7—H7A	0.9600
C5—C6	1.373 (3)	C7—H7B	0.9600
C5—H5A	0.9300	C7—H7C	0.9600
C6—C1	1.385 (3)		
			
C11—O2—C10	107.65 (16)	C2—C3—Cl	119.68 (19)
C13—C14—C10	126.5 (2)	C4—C3—Cl	119.3 (2)
C13—C14—H14A	116.7	C8—C11—O2	109.3 (2)
C10—C14—H14A	116.7	C8—C11—C7	134.4 (2)
C14—C13—C12	122.1 (2)	O2—C11—C7	116.28 (19)
C14—C13—H13A	118.9	C3—C2—C1	119.4 (2)
C12—C13—H13A	118.9	C3—C2—H2A	120.3
C9—C10—O2	108.0 (2)	C1—C2—H2A	120.3
C9—C10—C14	134.1 (2)	C10—C9—C8	107.5 (2)
O2—C10—C14	117.89 (17)	C10—C9—H9A	126.3
O1—C12—C13	121.0 (2)	C8—C9—H9A	126.3
O1—C12—C6	119.8 (2)	C11—C8—C9	107.6 (2)
C13—C12—C6	119.15 (18)	C11—C8—H8A	126.2
C4—C5—C6	121.3 (2)	C9—C8—H8A	126.2
C4—C5—H5A	119.4	C2—C1—C6	121.6 (2)
C6—C5—H5A	119.4	C2—C1—H1A	119.2
C5—C6—C1	117.3 (2)	C6—C1—H1A	119.2
C5—C6—C12	123.70 (19)	C11—C7—H7A	109.5
C1—C6—C12	119.0 (2)	C11—C7—H7B	109.5
C3—C4—C5	119.4 (2)	H7A—C7—H7B	109.5
C3—C4—H4A	120.3	C11—C7—H7C	109.5
C5—C4—H4A	120.3	H7A—C7—H7C	109.5
C2—C3—C4	121.0 (2)	H7B—C7—H7C	109.5

### Hydrogen-bond geometry (Å, °)

**Table:**

*D*—H···*A*	*D*—H	H···*A*	*D*···*A*	*D*—H···*A*
C9—H9A···O1^i^	0.93	2.53	3.380 (4)	153
C14—H14A···O1	0.93	2.47	2.795 (2)	100

Symmetry codes: (i) −*x*, −*y*, −*z*.
